# Is Two Better Than Three? A Systematic Review of Two-bag Intravenous N-acetylcysteine Regimens for Acetaminophen Poisoning

**DOI:** 10.5811/westjem.59099

**Published:** 2023-09-25

**Authors:** Jon B. Cole, Carrie L. Oakland, Samantha C. Lee, Kelly A. Considine, Maria I. Rudis, Alison L. Swanson, Travis D. Olives

**Affiliations:** * Hennepin Healthcare, Department of Emergency Medicine, Minneapolis, Minnesota; † University of Minnesota Medical School, Department of Emergency Medicine, Minneapolis, Minnesota; ‡ Minnesota Poison Control System, Minneapolis, Minnesota; § Hennepin Healthcare, Medical Intensive Care Unit, Department of Pharmacy, Minneapolis, Minnesota; ∥ Mayo Clinic, Department of Pharmacy, Rochester, Minnesota; ¶ Mayo Clinic, Department of Emergency Medicine, Rochester, Minnesota; # Children’s Hospitals and Clinics of Minnesota, Department of Pharmacy, Minneapolis, Minnesota

## Abstract

**Introduction:**

Acetaminophen poisoning is commonly treated by emergency physicians. First-line therapy is N-acetylcysteine (NAC), traditionally administered intravenously via a US Food and Drug Administration (FDA)-approved three-bag protocol in which each bag has a unique concentration and infusion duration. Recently, simplified, off-label two-bag NAC infusion protocols have become more common. The purpose of this review is to summarize the effectiveness and safety of two-bag NAC.

**Methods:**

We undertook a comprehensive search of PubMed, EMBASE, and MEDLINE from inception to December 13, 2022, for articles describing human acetaminophen poisonings treated with two-bag NAC, defined as any regimen involving two discrete infusions in two separate bags. Outcomes included effectiveness (measured by incidence of liver injury); incidence of non-allergic anaphylactoid reactions (NAAR); gastrointestinal, cutaneous, and systemic reactions; treatments for NAARs; incidence of NAC-related medication errors; and delays or interruptions in NAC administration.

**Results:**

Twelve articles met final inclusion, 10 of which compared two-bag NAC to the three-bag regimen. Nine articles evaluated the two-bag/20-hour regimen, a simplified version of the FDA-approved three-bag regimen in which the traditional first and second bags are combined into a single four-hour infusion. Nine articles assessed comparative effectiveness of two-bag NAC in terms of liver injury, most commonly assessed for by incidence of hepatotoxicity (aspartate aminotransferase or alanine aminotransferase >1,000 international units per liter). No difference in liver injury was observed between two-bag and three-bag regimens. Of nine articles comparing incidence of NAARs, eight demonstrated statistically fewer NAARs with two-bag regimens, and one showed no difference. In seven articles evaluating treatment for NAARs (antihistamines, corticosteroids, epinephrine), all showed that patients received fewer medications for NAARs with two-bag NAC. Three articles evaluated NAC-related medication errors; two demonstrated no difference, while one study evaluating only children showed fewer errors with two-bag NAC. Two studies evaluated delays and/or interruptions in NAC infusions; both favored two-bag NAC.

**Conclusion:**

For patients with acetaminophen poisoning, two-bag NAC regimens appear to have similar outcomes to the traditional three-bag regimen in terms of liver injury. Two-bag NAC regimens are associated with fewer adverse events and fewer treatments for those events than the three-bag regimen and fewer interruptions in antidotal therapy.

## INTRODUCTION

Acetaminophen poisoning is frequently seen by emergency physicians in the United States and is commonly reported to US poison centers. In 2021, US poison centers advised in over 87,000 cases of acetaminophen poisoning.^1^ Morbidity and mortality from acetaminophen poisoning are substantial. In the National Poison Data System (NPDS)—the national database owned and managed by America’s Poison Centers (formerly known as the American Association of Poison Control Centers), containing data from all 55 accredited US poison centers—acetaminophen was the most common substance associated with poisoning fatalities in 2021, contributing to 419 deaths.^1^ Acetaminophen is responsible for 50% of cases of acute liver failure (ALF) in the US each year, and acetaminophen-associated ALF accounts for approximately 7% of US liver transplants annually.^2^ ^,^ ^3^

N-acetylcysteine (NAC) has been the treatment of choice for acetaminophen poisoning for over four decades.^4^ ^,^ ^5^ Originally developed as an oral antidote, NAC is now most commonly administered via the intravenous (IV) route after its approval by the US Food and Drug Administration (FDA) in 2004.^5^ In the 2021 NPDS Annual Report, 29,377 patients received IV NAC, while only 1,909 received NAC via the oral route.^1^ Controversy remains, however, on the optimal IV NAC regimen. The FDA-approved IV NAC regimen involves administering 300 milligrams per kilogram (mg/kg) of IV NAC over 21 hours via three separate IV infusion bags, each with its own unique concentration and infusion rate (Table 1). While this regimen is time-tested, it leads to interruptions in antidote infusion and is associated with dosing errors.^6^ In addition, non-allergic anaphylactoid reactions (NAAR) frequently occur as a function of the large NAC dose administered in the first bag of the traditional protocol (Table 1).^7^

**Table 1.** Comparison of traditional three-bag intravenous N-acetylcysteine (NAC) regimen with two-bag NAC regimens.

Traditional 3-bag FDA-approved regimen (“Prescott protocol”)	Bag 1 (administered over 15–60 minutes)	Bag 2 (administered over 4 hours)	Bag 3 (administered over 16 hours)
Dose	150 mg/kg in 200 mL D5W	50 mg/kg in 500 mL D5W	100 mg/kg NAC in 1,000 mL D5W
Simplified 2-bag, 20-hour regimen	Bag 1 (administered over 4 hours)		Bag 2 (administered over 16 hours)
Dose	200 mg/kg in 500 mL D5W		100 mg/kg in 1,000 mL D5W
SNAP^*^ 12-hour IV NAC regimen	Bag 1 (administered over 2 hours)		Bag 2 (administered over 10 hours)
Dose	100 mg/kg in 200 mL D5W		200 mg/kg in 1,000 mL D5W

*IV*, intravenous; *NAC*, N-acetylcysteine; *FDA*, US Food and Drug Administration; *mL*, milliliter; *D5W*, dextrose 5% in sterile water; *mg*, milligram; *kg*, kilogram; *SNAP*, Scottish and Newcastle Antiemetic Pre-treatment for Paracetamol Poisoning, used in the United Kingdom with a unique treatment threshold (four-hour [APAP] = 100 mcg/mL nomogram line) compared to the United States (four-hour [APAP] = 150 mcg/mL nomogram line).

Over the past decade, evidence has emerged that a simplified two-bag IV NAC regimen is both safe and effective.^8^ ^–^ ^12^ A two-bag regimen is appealing as it may minimize interruptions in care, medication errors, and the incidence of dose-related NAARs.^7^ The traditional three-bag regimen, developed by Prescott and colleagues and first reported in 1977, involves a large initial bolus (150 mg/kg) of IV NAC over the first 15–60 minutes of treatment (which is when NAARs typically occur), whereas two-bag regimens generally extend the initial bolus of NAC over multiple hours (Table 1).^7^ ^,^ ^13^ Since NAARs are typically dose-related, reducing the infusion rate from the initial 150 mg/kg bolus in the traditional three-bag protocol may contribute to a reduction in NAARs. Multiple two-bag regimens have been studied, but an up-to-date summary of the evidence supporting their use is lacking. The purpose of this report was to review and summarize the effectiveness and safety of two-bag NAC regimens for acetaminophen poisoning. In the interest of precise pharmacologic nomenclature, we defined “two-bag NAC regimens” as any NAC regimen involving two discrete infusions in two separate bags (Table 1).^14^ Regimens involving a single bag of NAC with the rate adjusted at various times were not included for analysis.

## METHODS

### Search Strategy

Three searches were undertaken. The first search, performed by the primary author (JBC) on December 13, 2022, duplicated a previously published search strategy by searching PubMed using the following terms: (((Acetylcysteine) OR (NAC) OR (n-acetylcysteine)) AND ((novel) OR (alternative) OR (simplified) OR (off-label))) AND (overdose).^15^ The references of relevant articles were also reviewed by JBC for inclusion.

**Figure 1.**
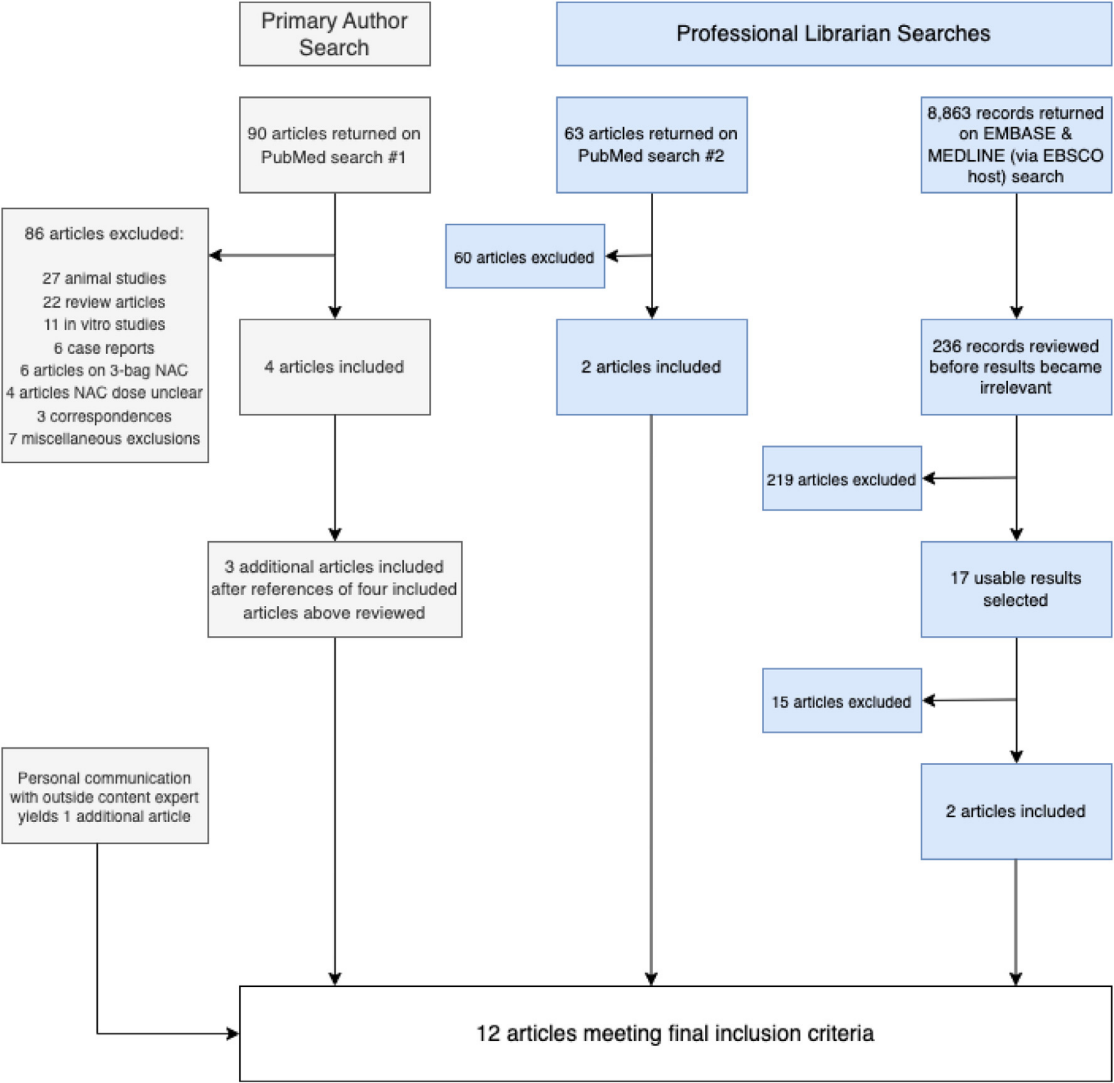
Screening process for article inclusion. *NAC*, N-acetylcysteine.

To ensure all relevant articles were included, we consulted a professional research librarian who performed two additional searches. First, PubMed was searched on December 14, 2022, using the following terms: (acetylcysteine) AND (acetaminophen poisoning) AND (safety). Second, a comprehensive search for English language articles was conducted using the EMBASE and MEDLINE libraries (separately, via EBSCOhost) (EBSCO Information Services, Ipswich, MA) from inception through December 14, 2022. The librarian crafted a search strategy to cover synonymous terms and phrases to retrieve pertinent articles related to human acetaminophen poisoning and NAC. The search strategy included the keywords noted above. Last, an outside expert in acetaminophen poisoning was also contacted to ensure the three searches returned all relevant articles. The complete search strategy is outlined in Figure 1.

### Inclusion Criteria

We sought to include articles containing data solely in human acetaminophen poisonings treated with two-bag NAC infusions. Editorials, commentaries, letters, case reports, and laboratory or animal data were excluded, as were articles on one- or three-bag NAC infusions. A single, board-certified emergency physician and medical toxicologist, working independently, reviewed the articles for inclusion and collected data from the articles. No automated tools were used.

### Outcomes

Outcomes of interest assessed for included effectiveness (measured by liver injury), incidence of NAARs (gastrointestinal, cutaneous, and systemic), medications used to treat NAARs, incidence of medication errors, and delays or interruptions in NAC administration. Effects were measured in absolute differences, odds ratios, and number needed to treat (NNT) as reported by the authors. When not reported, NNT was calculated from raw data in the articles. Similarly, we manually calculated unadjusted odds ratios with 95% confidence intervals for NAARs based on data from the included articles (if available), and from this a forest plot was generated to better define the reported effect of two-bag vs three-bag NAC regimens on NAARs.

## RESULTS

After initial searches and exclusion of irrelevant references (Figure 1), 11 articles met final inclusion criteria. Consultation with an outside expert yielded one additional article leaving 12 articles for final inclusion (Table 2), 10 of which compared 2-bag NAC regimens to the 3-bag regimen and two single-arm observational studies.^10^ ^,^ ^16^ Nine articles evaluated the 2-bag/20-hour regimen, a simplified version of the FDA-approved 3-bag regimen in which the traditional first and second bags are combined into a single four-hour 200 mg/kg infusion (Table 1).^9^ ^–^ ^12^ ^,^ ^17^ ^–^ ^21^ Two articles evaluated the Scottish and Newcastle Anti-emetic Pre-treatment for Paracetamol Poisoning (SNAP) protocol (Table 1).^8^ ^,^ ^22^ A single case series of 40 children evaluated a unique regimen not elsewhere reported.^16^

Seven articles evaluated the incidence of NAARs as the primary outcome.^8^ ^–^ ^10^ ^,^ ^17^ ^–^ ^19^ ^,^ ^21^ Three studies evaluated the incidence of hepatotoxicity as the primary outcome.^11^ ^,^ ^20^ ^,^ ^22^ One study each evaluated delays in treatment and serum sodium as the primary outcome.^12^ ^,^ ^16^ (Table 2) Nine articles assessed comparative effectiveness of two-bag NAC in terms of liver injury; liver injury was most commonly assessed for by incidence of hepatotoxicity (aspartate aminotransferase or alanine aminotransferase >1,000 international units per liter).^8^ ^,^ ^9^ ^,^ ^11^ ^,^ ^12^ ^,^ ^17^ ^,^ ^18^ ^,^ ^20^ ^–^ ^22^ In all nine articles no difference in liver injury was observed between groups; in two articles, subgroup analyses favored the two-bag regimen.^12^ ^,^ ^21^

**Table 2.** Evidence for improved outcomes with two-bag intravenous N-acetylcysteine regimens.

				Outcomes			
Study (Total n = 14,618)	Study type	2-bag NAC regimen studied	Primary outcome	NAARs	Hepatotoxicity	Dosing errors	Delays in treatment
Studies evaluating NAARs as primary outcome							
Bateman et al. 2014 (“SNAP Trial”)^8^ (n = 217)	RCT (adults)	SNAP (n = 108) vs. Prescott Protocol (n = 109)	Reduction in vomiting (including retching or need for antiemetics) in first 2 hours (36.1% vs. 65.1%; aOR = 0.26, [97.5% CI: 0.13–0.52], NNT = 3)	Reduction in clinically relevant severe^*^ NAARs (4.6% vs. 31%; aOR = 0.23, [97.5% CI: 0.12–0.43], NNT = 4)	No difference in 50% increase in ALT between the groups (12.9% vs. 9%; aOR = 0.60, [97.5% CI: 0.20–1.83])	NA	NA
Wong & Graudins. 2016^9^ (n = 599)	Retrospective cohort	2-bag/20-hours (n = 210) vs. Prescott Protocol (n = 389)	Fewer overall NAARs (4.3% vs 10%, OR = 2.5, [95% CI: 1.1–5.8], NNT = 18)	Fewer severe^**^ NAARs (0.5% vs. 1.8% [p < 0.01], NNT = 76)	No difference in hepatotoxicity (ALT >1,000 IU/L) (5.2% vs. 4.3%, p = 0.68, OR = 1.2, [95% CI: 0.55–2.63])	NA	NA
Isbister et al. 2016^10^ (n = 654)	Prospective observational (no comparison except to historical data)	Modified 2-bag based on ingestion time (n = 654)	Frequency of systemic hypersensitivity reactions = 8% (95% CI: 6–10%), lower than most previously published prospective studies of the Prescott protocol.	GI side effects similar to historic rates	16 patients had an ALT >1,000 IU/L	Four errors related to NAC; three of which involved incorrect infusion rate of bag #1.	NA
McNulty et al. 2018^17^ (n = 476)	Prospective data compared to historic controls	2-bag/20-hours (n = 163) vs. Prescott Protocol (n = 313)	Fewer NAARs; 14% vs 5% (difference: 9.4% [95% CI: 4.3–14.6%], p = 0.002, NNT = 11)	Fewer severe^***^ NAARs; 8% vs. 2% (difference: 6.1% [95% CI: 2.5 −9.8%], p = 0.007, NNT = 17) Fewer anti-allergy medications; 11% vs. 4% (difference: 6.9% [95% CI: 2.4–11.3%], p = 0.01, NNT = 15)	No difference in incidence of hepatotoxicity (4.8% vs. 3.7%)	NA	NA
Schmidt et al. 2018^18^ (n = 767)	Retrospective cohort	2-bag/20-hours (n = 493) vs. Prescott Protocol (n = 274)	Fewer NAARs; 17% vs 4% (difference = −12.8%, 95% CI:−17.6%–−8.0%, p < 0.01, NNT = 8)	Fewer severe NAARs (hypotension, edema, respiratory symptoms); 0.6% vs. 4% (p = 0.003, NNT = 30) Fewer cutaneous NAARs: 2% vs. 14% (p < 0.001, NNT = 9)	No difference in hepatotoxicity (4% vs. 4%, difference: 0%, 95% CI:−2.9%–3.0%)	Medication errors were rare (1%)	Fewer interruptions or delays in NAC; 5% vs. 12% (difference: 6.6% [95%CI: 2.2–10.9%], p = 0.002, NNT = 15)
Daoud et al. 2020^19^ (n = 4,315)	Retrospective cohort	2-bag/20-hours (n = 2,951) vs. Prescott Protocol (n = 1,364)	2-bag/20 hour NAC protocol associated with significantly fewer NAARs requiring treatment. (OR = 0.36 [95%CI: 0.28–0.46], 4% vs. 10.4%, NNT = 16)	Fewer life-threatening reactions (severe hypotension or airway-threatening angioedema): 0.6% vs. 0.14% Meta-analysis conducted revealed fewer NAARs with 2-bag regimens published to date.	NA	NA	NA
Sudanagunta et al. 2023^21^ (n = 243)	Retrospective cohort (children age <18 years)	2-bag/20-hours (n = 93) vs. Prescott Protocol (n = 150)	No overall difference in NAARs: 19% 2-bag vs. 23% 3-bag (p = 0.54)	NAARs Sub-analyses favoring 2-bag/20-hour protocol: Cutaneous NAARs: 2% vs. 10% (p = 0.02, NNT = 13) Fewer antihistamines administered for NAARs: 8% vs. 16% (p = 0.05, NNT = 13)	NA	Fewer NAC medication errors: 23% vs. 39% (p = 0.01, NNT = 7)	Majority of medication errors were due to timing defined as delays or pauses in NAC >1 hour
Studies evaluating hepatotoxicity as primary outcome							
Pettie et al. 2019^22^ (n = 3,340)	Prospective observational	SNAP (n = 1,852) vs. Prescott Protocol (n = 1,488)	No difference in liver injury, synthetic dysfunction, or hepatotoxicity (peak ALT >1,000 IU/L)	Fewer antihistamines given for NAARs; 11.0% vs. 2.0% (difference 9.0% [95% CI: 7.3–10.7], NNT = 10)	4.3% Prescott Protocol vs. 3.6% SNAP (difference −0.7%; 95% CI: −2.1–0.6%)	NA	NA
Wong et al. 2020 (“2NAC study”)^11^ (n = 2,211^*^) ^*^Single, acute ingestions included in non-inferiority analysis. 2,763 patients received NAC, however in 552 cases the dosing regimen was not specified	Retrospective cohort	2-bag/20-hours (n = 1,300) vs. Prescott Protocol (n = 911)	No difference in acute liver injury (peak ALT > 150 IU/L), regardless of time of presentation or peak [APAP].	Fewer NAARs; 7.1% vs. 1.3% (difference: 5.8% [95% CI: 4.0–7.6%], p < 0.0001, NNT = 18) Fewer GI side effects; 31% vs. 19% (p < 0.0001, NNT = 9)	No difference in hepatotoxicity (1.2% 2-bag vs. 1.6% 3-bag, difference: −0.4%, 95% CI: −1.75–0.91)	NA	NA
Syafira et al. 2022^20^ (n = 887)	Retrospective cohort (only patients receiving NAC within 8 hours of ingestion	2-bag/20-hours (n = 191) vs. Prescott Protocol (n = 696)	No difference in acute liver injury (peak aminotransferase > 150 IU/L): 1.6% 2-bag vs. 2.2 – 2.9% 3-bag (difference, 0.6%, OR 0.7 [95%CI: 0.2–2.6])	NA	No difference in hepatotoxicity (peak ALT >1,000 IU/L) between groups. Significantly higher proportion of patients with elevated aminotransferases (peak aminotransferase > 40 IU/L) in one of two 3-bag regimen cohorts: 14.8% vs. 3.7% (difference, 11.1%, OR = 0.2 [95%CI: 0.01–0.5], NNT = 10)	NA	NA
Studies evaluating delays in treatment as primary outcome							
O’Callaghan et al. 2022^12^ (n = 869)	Retrospective cohort	2-bag/20-hours (n = 598) vs. Prescott Protocol (n = 271)	Shorter median cumulative delays in NAC administration: 35 vs. 65 minutes (absolute difference: 30 minutes [95%CI: 20 – 33], p < 0.01)	Fewer GI side effects: 76% vs. 56% (p < 0.0001, NNT = 5) Fewer cutaneous NAARs: 4.2% vs. 10% (p < 0.0001, NNT = 18) Fewer systemic NAARs: 4.1% vs. 0.8% (p < 0.001, NNT = 31)	Higher median ALT (40 IU/L vs. 19 IU/L) in patients receiving the 3-bag regimen with delays > 3 hours	NA	Delays inNAC administration > 1 hour less common with 2-bag (31% vs. 51%, p < 0.01, NNT = 5)
Studies evaluating serum sodium levels as primary outcome							
Oakley et al. 2011^16^ (n = 40)	Case series (children ≤17 years)	150 mg/kg over one hour, then 10 mg/kg/hr for 20 hours (n = 40)	Serum sodium remained in normal range using 0.45% saline as the NAC diluent rather than D5W to prevent iatrogenic hyponatremia in children.	18 patients (49%) had any adverse reaction.	NA	NA	NA

^*^ Clinically significant NAARs: requiring drug treatment or interruption of NAC infusion.

^**^ Severe NAARs: angioedema, bronchospasm, or hypotension.

^***^ Severe NAARs: hypotension, dyspnea, swelling.

*RCT*, randomized controlled trial; *SNAP*, Scottish and Newcastle Antiemetic Pre-treatment for Paracetamol Poisoning; *NAC*, N-acetylcysteine; *OR*, odds ratio; *aOR*, adjusted odds ratio; *CI*, confidence interval; *NNT*, number needed to treat; *NAARs*, non-allergic anaphylactoid reactions, which in some studies include both gastrointestinal and systemic effects; *ALT*, alanine aminotransferase; *GI*, gastrointestinal; *D5W*, dextrose 5% in sterile water.

**Figure 2.**
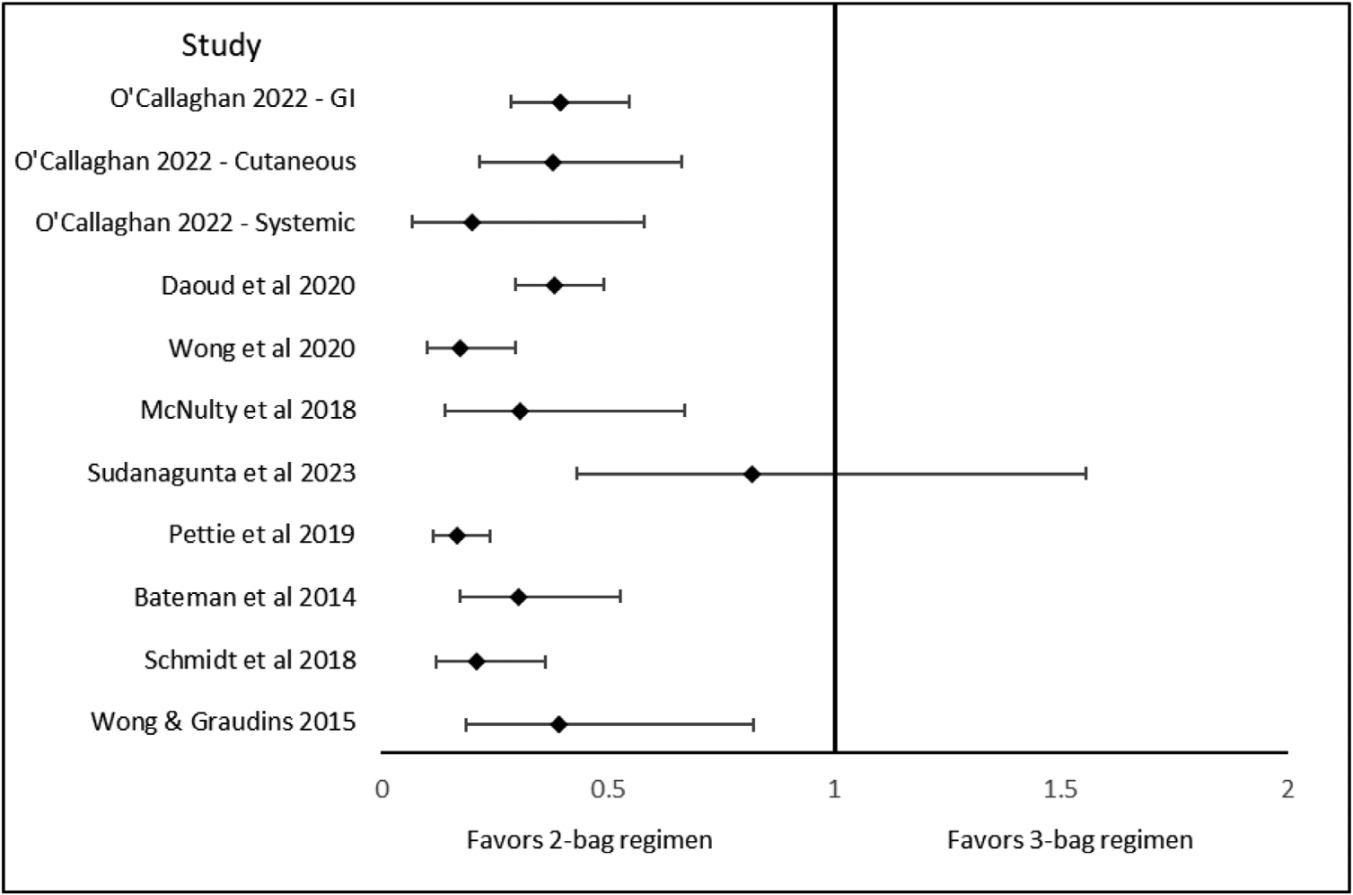
Forest plot of non-allergic anaphylactoid reactions (NAARs) reported in studies that compare two-bag to three-bag N-acetylcysteine infusions for acetaminophen poisoning. Aggregate data for NAARs are displayed for all studies with the exception of O’Callaghan et al, as that study’s data was reported by the individual organ system.

Nine articles assessed comparative effectiveness of two-bag NAC regarding incidence of NAARs (Table 3).^8^ ^,^ ^9^ ^,^ ^11^ ^,^ ^12^ ^,^ ^17^ ^–^ ^19^ ^,^ ^21^ ^,^ ^22^ The definition of NAARs varied between studies; each study’s NAARs definition is displayed in Table 3. All but one article demonstrated statistically fewer NAARs with two-bag regimens.^21^ The single article demonstrating no difference in NAARs between two-bag and three-bag regimens studied 243 children (age <18 years) and reported fewer cutaneous NAARs associated with two-bag NAC in subgroup analysis.^21^ Reductions in cutaneous and systemic NAARs were more common than reductions in gastrointestinal (GI) NAARs (Table 3). Eight comparative studies evaluated GI NAARs, three favored two-bag NAC while five showed no difference when comparing two-bag and three-bag regimens.^8^ ^,^ ^9^ ^,^ ^11^ ^,^ ^12^ ^,^ ^17^ ^–^ ^19^ ^,^ ^21^ In contrast, seven studies evaluated cutaneous NAARs; all but one favored two-bag NAC regimens.^9^ ^,^ ^11^ ^,^ ^12^ ^,^ ^17^ ^–^ ^19^ ^,^ ^21^ ^,^ ^22^ Seven articles evaluated use of anti-allergy medications to treat NAARs (antihistamines, corticosteroids, and epinephrine); all seven studies favored two-bag NAC regimens.^9^ ^,^ ^12^ ^,^ ^17^ ^–^ ^19^ ^,^ ^21^ ^,^ ^22^ Four studies reported granular data on the use of anti-allergy medications; all four studies favored two-bag NAC.^9^ ^,^ ^19^ ^,^ ^21^ ^,^ ^2^ A summary of calculated unadjusted odds ratios with 95% confidence intervals for NAARs, comparing two-bag and three-bag regimens, is displayed as a forest plot in Figure 2.

**Table 3.** Studies evaluating non-allergic anaphylactoid reactions in two-bag intravenous N-acetylcysteine regimens.

				Specific NAARs			
Study (Total N = 13,731)	Study type	2-bag NAC regimen studied	NAARs definitions	GI effects	Total NAARs and/or systemic NAARs	Skin-only reactions	Anti-allergy medications administered as treatment
Studies with comparative data evaluating NAARs as primary outcome							
Bateman et al 2014 (“SNAP Trial”)^8^ (n = 217)	RCT (adults)	SNAP (n = 108) vs. Prescott Protocol (n = 109)	Reduction in vomiting (including retching or need for antiemetics) in first 2 hoursAnaphylactoid reactions, defined as need for treatment or NAC interruption; self-reported flushing, itchy skin, rash, chest pain, dyspnea, wheezing, tongue/lip swelling	Reduction in vomiting (including retching or need for antiemetics) in first 2 hours (36.1% vs. 65.1%; aOR = 0.26, [97.5% CI: 0.13–0.52], NNT = 3)	Reduction in clinically relevant severe^*^ NAARs (4.6% vs. 31%; aOR = 0.23, [97.5% CI: 0.12–0.43], NNT = 4) Fewer total anaphylactoid reactions (54% vs. 75%)	NA	NA
Wong and Graudins. 2016^9^ (n = 599)	Retrospective cohort	2-bag/20-hours (n = 210) vs. Prescott Protocol (n = 389)	NAARs classified into cutaneous (flushing, rash, urticaria. Wheals, itch) and more severe reactions including respiratory symptoms (bronchospasm, wheeze, dyspnea) and angioedema or cardiovascular instability (i.e., hypotension) GI symptoms included nausea or vomiting.	No difference in gastrointestinal side effects (2-bag = 41%, 3-bag = 39%)	Fewer severe^**^ NAARs (0.5% vs. 1.8% [p < 0.01], NNT = 76) Fewer overall NAARs (4.3% vs 10%, OR = 2.5, [95% CI: 1.1–5.8], NNT = 18)	Fewer reactions (8%) with 2-bag compared to 3-bag (33%)	Assessed for but not reported
McNulty et al 2018^17^ (n = 476)	Prospective data compared to historic controls	2-bag/20-hours (n = 163) vs. Prescott Protocol (n = 313)	Cohorts compared for adverse reactions to NAC (not further defined), or use of anti-allergy medications	No difference in gastrointestinal side effects (3-bag = 37%, 2-bag = 31%).	Fewer NAARs; 14% vs 5% (difference: 9.4% [95% CI: 4.3 – 14.6%], p = 0.002, NNT = 11) Fewer severe^***^ NAARs; 8% vs. 2% (difference: 6.1% [95% CI: 2.5 −9.8%], p = 0.007, NNT = 17)	No difference in skin-only reactions (3-bag = 6%, 2-bag = 3%, p = 0.12)	Fewer anti-allergy medications; 11% vs. 4% (difference: 6.9% [95% CI: 2.4 – 11.3%], p = 0.01, NNT = 15)
Schmidt et al 2018^18^ (n = 767)	Retrospective cohort	2-bag/20-hours (n = 493) vs. Prescott Protocol (n = 274)	Cutaneous (flushing, rash, urticaria, wheals, itch), severe reactions (bronchospasm, wheeze, dyspnea) and angioedema or cardiovascular instability (i.e., hypotension). Intensity of symptoms rated as mild (aware of signs/symptoms but easily tolerated), moderate (discomfort to interfere with usual activity), severe (incapacitating with inability to work or do usual activity) or unknown.	No difference in gastrointestinal side effects (3-bag = 1%, 2-bag = 0%).	Fewer NAARs; 17% vs 4% (difference = −12.8%, 95% CI:−17.6% – −8.0%, p < 0.01, NNT = 8) Fewer severe NAARs (hypotension, edema, respiratory symptoms); 0.6% vs. 4% (p = 0.003, NNT = 30)	Fewer cutaneous NAARs: 2% vs. 14% (p < 0.001, NNT = 9)	Assessed for but not reported
Daoud et al 2020^19^ (n = 4,315)	Retrospective cohort	2-bag/20-hours (n = 2,951) vs. Prescott Protocol (n = 1,364)	Defined as a reaction requiring treatment with IV antihistamine and/or glucocorticoids	GI effects rare for both groups, no difference found: 2-bag = 0.24%, 3-bag = 0.51%, p = 0.14	2-bag/20 hour NAC protocol associated with significantly fewer NAARs requiring treatment. (OR = 0.36 [95%CI: 0.28 – 0.46], 4% vs. 10.4%, NNT = 16) Fewer life-threatening reactions (severe hypotension or airway-threatening angioedema): 0.6% vs. 0.14%	Fewer cutaneous reactions with 2-bag (2.9%) vs. 3-bag (7.7%), p < 0.0001	Fewer medications needed to treat NAARs with 2-bag (6.9%) vs. 3-bag (19.7%), p < 0.0001
Sudanagunta et al 2023^21^ (n = 243)	Retrospective cohort (children age <18 years)	2-bag/20-hours (n = 93) vs. Prescott Protocol (n = 150)	NAARs divided into 4 organ systems (cutaneous, cardiovascular, GI, respiratory), keywords searched for in chart (e.g., urticaria, wheal, edema, bronchospasm, wheeze, nausea, vomiting, hypotension)	No difference in GI symptoms: (3-bag = 11%, 2-bag = 11%)	No overall difference in NAARs: 19% 2-bag vs. 23% 3-bag (p = 0.54)	Fewer cutaneous NAARs: 2% vs. 10% (p = 0.02, NNT = 13)	Fewer antihistamines administered for NAARs: 8% vs. 16% (p = 0.05, NNT = 13)
Studies with comparative data evaluating NAARs as a secondary outcome							
Pettie et al 2019^22^ (n = 3,340)	Prospective observational	SNAP (n = 1,852) vs. Prescott Protocol (n = 1,488)	Antihistamine prescribing was used to estimate the rate of anaphylactoid reactions.	NA	Fewer antihistamines given for NAARs; 11.0% vs. 2.0% (ARR = 9.0% [95% CI: 7.3–10.7], NNT = 10)	NA	See total NAARs; sub-analysis of 37 patients receiving both regimens for multiple overdoses (198 admissions, 81 received 3-bag, 117 received SNAP), found NAARs occurred in 6.2% of 3-bag admissions and in 0.9% of SNAP admissions (ARR = 5.3%, 95% CI: 0.1–12.8%)
Wong et al 2020 (“2NAC study”)^11^ (n = 2,211^*^) ^*^Single, acute ingestions included in non-inferiority analysis. 2,763 patients got NAC, however in 552 cases the dosing regimen was not specified	Retrospective cohort	2-bag/20-hours (n = 1,300) vs. Prescott Protocol (n = 911)	NAARs classified into cutaneous (flushing, rash, urticaria, itch), more severe reactions (bronchospasm, wheeze, dyspnea, angioedema, cardiovascular instability [i.e., hypotension]), and GI symptoms (nausea, vomiting, or both)	Fewer gastrointestinal side effects; 31% vs. 19% (p<0.0001, NNT = 9)	Fewer Cutaneous and Systemic NAARs: combined data 7.1% vs. 1.3% (difference: 5.8% [95% CI: 4.0–7.6%], p < 0.0001, NNT = 18)	Fewer Cutaneous NAARs: (2-bag, 1.1% vs. 3-bag, 6.4%)	NA
O’Callaghan et al 2022^12^ (n = 869)	Retrospective cohort	2-bag/20-hours (n = 598) vs. Prescott Protocol (n = 271)	Adverse reactions assessed in three categories: gastrointestinal (nausea/vomiting/retching), cutaneous (rash/itch/flushing), and systemic (bronchospasm/hypotension/angioedema or administration of medications)	Fewer gastrointestinal side effects: 76% vs. 56% (p < 0.0001, NNT = 5)	Fewer systemic NAARs: 4.1% vs. 0.8% (p < 0.001, NNT = 31)	Fewer cutaneous NAARs: 4.2% vs. 10% (p < 0.0001, NNT = 18)	Assessed for but not reported individually
Single-arm studies evaluating non-allergic anaphylactoid reactions.							
Oakley et al 2011^16^ (n = 40)	Case series (children ≤17 years)	150 mg/kg over one hour, then 10 mg/kg/hr for 20 hours (n = 40)	Not defined	11 patients (30%) had vomiting.	18 patients (49%) had any adverse reaction. One patient each (2.5%) had breathlessness, abdominal pain, and cough.	Four (11%) patients developed rash.	NA
Isbister et al 2016^10^ (n = 654)	Prospective observational (no comparison except to historical data)	Modified 2-bag based on ingestion time (n = 654)	Primary outcome was proportion of patients with adverse reactions, including only GI symptoms (not further defined) and the proportion with systemic hypersensitivity reactions, defined as either skin only or non-immune mediated anaphylaxis as defined by NIAID-FAAN. Severe anaphylaxis reactions were defined by hypotension or hypoxia	Gastrointestinal side effects similar to historic rates (26.5%)	Frequency of systemic hypersensitivity reactions = 8% (95% CI: 6–10%), lower than most previously published prospective studies of the Prescott Protocol. 0.5% had severe anaphylaxis	8% had skin-only reactions	NA

^*^ Clinically significant NAARs: requiring drug treatment or interruption of NAC infusion.

^**^ Severe NAARs: angioedema, bronchospasm, or hypotension.

^***^ Severe NAARs: hypotension, dyspnea, swelling.

*RCT*, randomized controlled trial; *SNAP*, Scottish and Newcastle Antiemetic Pre-treatment for Paracetamol Poisoning; *NAC* = N-acetylcysteine; *GI,* gastrointestinal, *OR*, odds ratio; *aOR*, adjusted odds ratio; CI, confidence interval; *NNT*, number needed to treat; *NAARs*, non-allergic anaphylactoid reactions, which in some studies include both gastrointestinal and systemic effects; *ALT*, alanine aminotransferase, *ARR*, absolute risk reduction, *NIAID-FAAN*, National Institute of Allergy and Infectious Diseases/Food Allergy and Anaphylaxis Network.

Three articles evaluated medication errors related to NAC; two demonstrated no difference, while one study evaluating only children showed fewer errors with two-bag NAC.^9^ ^,^ ^18^ ^,^ ^21^ Two studies evaluated delays and/or interruptions in NAC infusions; both favored two-bag NAC.^12^ ^,^ ^18^

## DISCUSSION

This systematic review demonstrates that two-bag NAC regimens have similar and, in some studies, non-inferior outcomes to the traditional three-bag regimen in terms of liver injury from acetaminophen poisoning while resulting in fewer adverse reactions, fewer treatments for adverse reactions, and fewer delays or interruptions in NAC infusions. Two-bag NAC regimens are associated with fewer adverse events, including cutaneous (eg, flushing, itching) and systemic (eg, bronchospasm, hypotension, angioedema) reactions.^8^ ^–^ ^12^ ^,^ ^17^ ^–^ ^19^ ^,^ ^22^ Fewer GI side effects were observed with two-bag NAC as well, although this finding was less common.^8^ ^,^ ^11^ ^,^ ^12^ Two-bag NAC infusion regimens may also result in fewer medication errors. Of the published two-bag regimens, the two-bag/20-hour regimen that combines bags one and two of the traditional FDA-approved three-bag regimen is the most studied (Table 1).

All but one study with comparative data favored two-bag NAC regimens over the traditional three-bag Prescott protocol, and the single negative study evaluated only children, was relatively small in terms of enrollment, and did favor two-bag NAC when considering both cutaneous NAARs and anti-allergy medications administered.^21^ Although NAARs definitions varied from study to study (Table 3), a decrease in both mild and severe effects was routinely associated with two-bag NAC regimens. For instance, the Scottish and Newcastle Antiemetic Pre-treatment for Paracetamol Poisoning (SNAP) trial (Table 1) demonstrated a reduction in severe NAARs from 31% to 4.6% when a two-bag protocol was used.^8^ Follow-up data from implementation of the SNAP protocol saw a reduction in antihistamine use from 11% with the traditional three-bag protocol to 2% when SNAP was used in a study of 3,340 patients.^22^ Similarly for the two-bag/20-hour protocol, prospectively collected data showed this protocol’s implementation was associated with a reduction in severe NAARs from 8% with the three-bag regimen to 2%.^17^ Multicenter implementation data evaluating the two-bag/20-hour protocol showed a drop in overall NAARs from 7.1% with the three-bag regimen to 1.3%.^11^ Significant reductions in GI (76% to 56%), cutaneous (10% to 4.2%), and systemic (4.1% to 0.8%) NAARs were also seen after implementation of the two-bag/20-hour protocol.^12^

Because of the advantages noted above, many toxicologists and poison centers have adopted a two-bag NAC regimen as their first-line therapy for treating acetaminophen poisoning.^4^ ^,^ ^11^ ^,^ ^14^ ^,^ ^19^ ^,^ ^22^ ^,^ ^23^ For practice in the United States, when considering a two-bag NAC regimen, a logical choice is the two-bag/20-hour protocol. While data on the SNAP protocol is robust, his data was generated in the United Kingdom, where the treatment threshold for NAC in acute acetaminophen poisoning is typically based upon a nomogram with a treatment line set at a four-hour acetaminophen concentration of 100 micrograms per milliliter (mcg/mL).^8^ ^,^ ^22^ In comparison, in the US, a 150 mcg/mL threshold is commonly used, making the SNAP data less generalizable to US practice. The 2-bag/20-hour protocol is now a reliable international standard; it is now the first-line recommended regimen in Australia, New Zealand, Denmark, and Sweden.^19^ ^,^ ^24^ ^,^ ^25^ We also believe the two-bag/20-hour regimen has the most robust body of supporting evidence, as its introduction in multiple studies results in consistent reductions in NAARs. To put this in clinical context, in 2021 987 patients reported to our regional poison center received IV NAC for acetaminophen poisoning. The NNT to reduce the incidence of various NAARs for the two-bag/20-hour regimen (Tables 2 and 3) is as low as five. Using a more conservative NNT of 11 from one study, if the two-bag/20-hour regimen were applied to our population of 987 patients, almost 90 fewer people would experience NAARs in one year.^17^

The adoption of the two-bag/20-hour protocol has several advantages for emergency physicians at the local level. Most IV NAC in the US is started in emergency departments (ED).^26^ Beyond the obvious advantage of a simpler regimen with half the number of additional orders to place, fewer orders for pharmacy departments to process and bags to prepare, and fewer bags for nurses to hang, the two-bag/20-hour protocol is associated with a significant reduction in NAARs (as noted above). Most NAARs with the traditional three-bag regimen occur in the first hour or two of the infusion, while the patient is in the ED.^7^ A reduction in NAARs during this time period not only results in a better patient experience, it results in fewer interruptions for the emergency physician, nurse, and pharmacist to attend to a patient’s adverse reaction, including reactions that require additional medication administration such as antihistamines, antiemetics, corticosteroids, and even epinephrine. Particularly important for the practice of emergency medicine, any systemwide change to the two-bag/20-hour NAC regimen will disproportionately affect the emergency medicine team, as all the changes from the traditional three-bag regimen occur in the first four hours of the infusion when the patient is likely to still be in the ED. Appropriate resource utilization and decreasing unnecessary treatments and interventions are increasingly important as ED boarding has become more common since the COVID-19 pandemic.^27^ Regardless, for US emergency physicians adopting a two-bag NAC regimen, poison centers remain available 24 hours a day, 365 days a year, at 1-800-222-1222 to answer questions regarding modified NAC protocols.

## LIMITATIONS

This review has several limitations. We searched only for English language articles. Our search may have been incomplete. For example, unlike some toxicologic reviews, we did not search academic meeting abstracts for data published only in abstract form, preferring to review only data that had undergone peer-review and was published in indexed journals.^28^ We also did not include editorials, commentaries, letters, or individual case reports. We excluded editorials, commentaries, and letters because they were unlikely to include original data. Individual case reports were excluded because claims about effectiveness and safety are difficult to infer from single cases, and because case reports focusing on two-bag NAC regimens are exceedingly rare. Nevertheless, it is possible that meaningful data was missed in any of these forms of articles that could have affected our results.

Two-bag regimens are not adequately studied in unusual or extreme circumstances, such as massive overdoses.^29^ The safety and effectiveness of two-bag NAC regimens in these uncommon circumstances are still understudied; however the same is true for the standard three-bag regimen. In large overdoses, such as overdoses of 30 grams or more, commensurate larger doses of NAC may be required, and consultation with a poison center or medical toxicologist is advised as tailored NAC dosing may be needed to prevent or treat liver injury.^30^

Additionally, we only evaluated two-bag NAC regimens. Data exists to support the use of a one-bag regimen in which the infusion rate of a single bag and concentration of NAC is changed at various points during treatment.^31^ ^–^ ^33^ While we understand the rationale for this unique approach, evaluation of one-bag regimens was not the purpose of our review.

Last, NAARs may have been inadequately documented in some of the studies we reviewed. The detection of adverse drug reactions is often under-reported in retrospective studies when compared with subsequent clinical trials.^34^^,^^35^ We suspect this may be the case with the present data. For example, GI side effect rates in the present studies range from 76% to <1%, suggesting they were under-reported in some studies, particularly those that are retrospective in nature. If GI side effects are poorly documented (or undocumented) in the medical records of study subjects, it may be difficult to detect a difference in nausea or vomiting after implementation of a two-bag NAC regimen. Such bias could lead to over- or under-estimating the effect of two-bag NAC regimens.

## CONCLUSION

For patients with acetaminophen poisoning, two-bag NAC regimens appear to have similar outcomes to the traditional three-bag regimen in terms of liver injury while resulting in fewer adverse reactions, fewer treatments for adverse reactions, and fewer delays or interruptions in NAC infusions. A two-bag infusion may also result in fewer medication errors. Of the published two-bag regimens, the two-bag/20-hour regimen that combines bags one and two of the traditional three-bag regimen is the most studied.
